# Obstetricians’ Attitudes Toward the Treatment of Extremely Preterm Infants in China

**DOI:** 10.1001/jamanetworkopen.2022.33511

**Published:** 2022-09-27

**Authors:** Tao Han, Dan Wang, Wenyu Xie, Changgen Liu, Qian Zhang, Zhichun Feng, Qiuping Li

**Affiliations:** 1 Department of Neonatology, Senior Department of Pediatrics, the Seventh Medical Center of Chinese PLA General Hospital, Beijing, China; 2 National Engineering Laboratory for Birth Defects Prevention and Control of Key Technology, Beijing, China; 3 Beijing Key Laboratory of Pediatric Organ Failure, Beijing, China; 4 The Second School of Clinical Medicine, Southern Medical University, Guangzhou, China

## Abstract

**Importance:**

Extremely preterm infants (EPIs) are at high risk of mortality and disability, and whether obstetricians are active in treating EPIs is directly related to whether EPIs can get an opportunity to enter the neonatal intensive care unit (NICU) for treatment. A greater understanding of the attitudes of obstetricians toward the treatment of EPIs is needed to improve treatment of this population and optimize pediatric health policies and services.

**Objective:**

To clarify the attitudes of obstetricians toward the treatment of EPIs in China.

**Design, Setting, and Participants:**

A cross-sectional, online, questionnaire-based survey was conducted among obstetricians from various provinces and cities in China through the *wenjuanxing* application (a survey platform) between June and July 2021. The questionnaire included characteristics of the participants, their experiences in delivering EPIs, their attitudes toward EPI resuscitation, and their opinions about the cutoff for providing full care to preterm infants in China. Possible factors associated with their attitudes toward the cutoff for providing full care to preterm infants were analyzed by logistic regression. Data analysis was performed from August 2021 to January 2022.

**Main Outcomes and Measures:**

The attitudes of obstetricians toward the resuscitation and treatment of EPIs, and the current cutoff for providing full care to premature infants.

**Results:**

A total of 2817 valid questionnaires were recovered from 30 provinces and cities in mainland China. Of the 2817 obstetricians (mean [SD] age, 41.76 [8.50] years) investigated, 156 (5.5%) were male, 2661 (94.5%) were female, 1339 (47.5%) were deputy chief physicians or chief physicians, 1662 (59.0%) were from tertiary hospitals, and 2048 (72.7%) had experience in delivering EPIs. The proportion of obstetricians who supported resuscitation of preterm infants was 19.1% (539 respondents) at a gestational age 24 weeks, 24.1% (679 respondents) at 25 weeks, 54.8% (1543 respondents) at 26 weeks, and 100.0% (2817 respondents) at 27 weeks. A total of 1326 obstetricians (47.1%) reported that it was common or very common for EPIs to be withdrawn from resuscitation and not to be admitted to the NICU for treatment. Regarding the treatment of EPIs with higher possibility of survival, 1621 obstetricians (57.5%) maintained that appropriate information should be communicated but their parents’ wishes should be respected. Most obstetricians (2051 respondents [72.8%]) maintained that 28 weeks was the cutoff for providing full care to preterm infants in China; 766 respondents (27.2%) thought of it as inappropriate, suggesting the cutoff should be less than 28 weeks. Logistic regression analysis showed that these differences were significantly associated with the professional titles of the obstetricians, geographical regions, hospital levels and types, whether they had any experience in delivering EPIs, and the total number of EPIs born yearly in their units.

**Conclusions and Relevance:**

In this cross-sectional study, most Chinese obstetricians maintained a conservative attitude toward the treatment of EPIs. It was very common for EPIs to be withdrawn from treatment without entering the NICU directly after birth. Most obstetricians maintained that 28 weeks’ gestational age should be the cutoff for providing full care to premature infants.

## Key Points

**Question:**

What are the attitudes of obstetricians toward the resuscitation and treatment of extremely preterm infants (EPIs) in China?

**Findings:**

In this cross-sectional study of 2817 obstetricians from 30 provinces and cities in mainland China in 2021, the proportion of obstetricians who supported resuscitation of preterm infants was 19.1% at a gestational age of 24 weeks, 24.1% at 25 weeks, 54.8% at 26 weeks, and 100.0% at 27 weeks. In addition, 27.2% of obstetricians suggested that the cutoff for providing full care to preterm infants in China should be adjusted further downward.

**Meaning:**

These findings suggest that most Chinese obstetricians maintain a conservative attitude toward the treatment of EPIs, which is mainly related to their concern about the poor prognosis.

## Teaser

This cross-sectional study examines the attitudes of obstetricians toward the treatment of extremely preterm infants in China.

## Introduction

Extremely preterm infants (EPIs) are premature infants with a gestational age of less than 28 weeks who are at high risk of mortality and disability because of their extremely immature organ development and the extreme difficulty in treating them.^1^ According to the 2013 World Health Organization Global Premature Infant Report,^2^ approximately 780 000 EPIs are born worldwide every year. These EPIs are a small part of the birth population, their treatment often requires a deal of medical resources, and treatment may be unsuccessful. Apart from the therapeutic cost, the treatment of EPIs also involves ethical and legal considerations.

At present, there are many ethical controversies over the lowest gestational age for the treatment of EPIs, even in developed countries and regions.^3,4^ In recent years, with the development of perinatal systems and technical advancements in the treatment of premature infants, both the short-term and long-term prognosis of EPIs, especially those over 24 weeks’ gestational age, have improved remarkably.^5,6^ At present, most developed countries tend to adopt a positive attitude toward the treatment of EPIs.^7^ A study^8^ from the US showed that, from 2013 to 2018, the survival rates were 10.9% for infants born alive at 22 weeks, 49.4% for those born at 23 weeks, and 94.0% for those born at 28 weeks. In addition, the percentage of infants for whom active treatment was implemented was 36.5% for those born at 22 weeks, 88.5% for those born at 23 weeks, 97.9% for those born at 24 weeks, and more than 99.0% for those born at 25 to 28 weeks. A Chinese small sample survey^9^ on the attitudes of neonatologists toward the active treatment of EPIs in 2017 showed that 21% of respondents would only resuscitate infants born at 28 weeks or later, indicating that the enthusiasm of treating EPIs in China is generally lower than that in developed countries.

Since the beginning of the 21st century, more neonatal intensive care units (NICUs) have been established throughout China, and more EPIs have been treated successfully in terms of both the survival rate and prognosis, especially in NICUs of large-scale tertiary hospitals, where the survival rate of EPIs over 24 weeks’ gestational age has reached a level comparable to that in developed countries.^10,11^ However, according to the guidelines for clinical diagnosis and treatment of preterm infants by the Obstetrics and Gynecology Branch of the Chinese Medical Association,^12^ the current cutoff for providing full care to preterm infants in China is gestational age 28 weeks. Because of the difficulty of treatment and the high incidence of complications after survival, preterm infants born before 28 weeks are not covered.^12^ Most EPIs were withdrawn from resuscitation in the delivery room, and, therefore, the proportion of them receiving active treatment in the NICU is very low.^13^ The treatment of EPIs has also been a medical controversy, causing serious medical disputes and social concerns. The treatment of EPIs highly depends on well-established perinatal systems, requiring concerted cooperation between obstetrics and pediatrics, especially on the part of the obstetrician. In China, although neonatologists are the leading force in EPI resuscitation, they are not always involved in prenatal communication with the parents. Obstetricians are important members of the resuscitation team, and they are direct communicators with the parents; therefore, their attitudes have great impact on the treatment decisions of the parents. To clarify the attitude of Chinese obstetricians toward the treatment of EPIs and their opinions about the cutoff for providing full care to preterm infants in China, we organized this national survey to provide information for improving the treatment of EPIs and optimizing pediatric health policies and services in future.

## Methods

This cross-sectional study was approved by the research ethics board of the Seventh Medical Center of Chinese PLA General Hospital (Beijing, China), with a waiver of informed consent from this ethics board because of the observational design of the study. This study follows the Strengthening the Reporting of Observational Studies in Epidemiology (STROBE) reporting guideline.

### Survey Respondents and Questionnaire Formulation

The survey respondents were obstetricians in China under the following inclusion criterion: obstetricians registered in public hospitals in mainland China. The questionnaire form was designed after repeated discussions and revisions by hospital management experts and senior neonatal and obstetric experts. The questionnaire consisted of 28 items, including characteristics of the participants, the annual delivery number of EPIs in their units, their attitudes toward the resuscitation of EPIs at different gestational ages, and their opinions about the cutoff for providing full care to preterm infants in China.

### Survey Time and Methods

The survey was conducted from June to July 2021. A questionnaire form was designed and distributed via the survey platform *wenjuanxing* among several WeChat groups composed of national obstetric experts in China, and then was forwarded to obstetrician groups in their provinces and cities for online survey. There were standard instructions for the questionnaire survey, and appointed people were assigned to be responsible for the distribution, recovery, and quality control of the questionnaires. The questionnaires were anonymous, and all questions in the questionnaire were required to be answered.

### Statistical Analysis

The results conforming to normal distribution are expressed as mean and SD, and categorical data are presented as frequencies and percentages. A multivariable logistic regression analysis was conducted to examine factors associated with obstetricians’ opinions about the current lowest gestational age for providing full care to preterm infants. The variables included in the model were as follows: gender, professional title, marital status, having children or not, region, working history, hospital level, hospital category, experience of delivering EPIs, and the annual delivery number of EPIs. All *P* values were 2-sided, and significance was set at *P* < .05. The survey data were collected and statistically analyzed by use SPSS statistical software version 26.0 (IBM). Data analysis was performed from August 2021 to January 2022.

## Results

### Characteristics of the Respondents

The survey included 2817 obstetricians (156 male [5.5%] and 2661 female [94.5%]) with a mean (SD) age of 41.76 (8.50) years and a mean (SD) working history of 17.73 (9.48) years. Of them, 1662 (59.0%) were from tertiary hospitals, 1339 (47.5%) were deputy chief physicians or chief physicians, 2048 (72.7%) had experience in delivering EPIs, and 1612 (57.2%) were from hospitals where the annual delivery number of EPIs was no more than 10. Characteristics of the included obstetricians are shown in Table 1.

**Table 1.** Characteristics of the Respondents

Characteristics	Respondents, No. (%) (N = 2817)
Gender	
Male	156 (5.5)
Female	2661 (94.5)
Marital status	
Married	2557 (90.8)
Unmarried	215 (7.6)
Others	45 (1.6)
Have children	
Yes	2488 (88.3)
No	329 (11.7)
Ethnicity	
Han	2503 (88.9)
Any other ethnicity	314 (11.1)
Region	
Eastern	870 (30.9)
Central	679 (24.1)
Western	1268 (45.0)
Professional title	
Resident physician	557 (19.8)
Attending physician	921 (32.7)
Deputy chief	904 (32.1)
Chief	435 (15.4)
Hospital level	
Tertiary	1662 (59.0)
Secondary	1035 (36.7)
Primary	120 (4.3)
Hospital category	
General	2033 (72.2)
Maternal and child health care	784 (27.8)
Working history, y	
<5	236 (8.4)
5-9	388 (13.8)
10-19	904 (32.1)
20-29	894 (31.7)
≥30	395 (14.0)
Experience in delivering EPIs	
Yes	2048 (72.7)
No	769 (27.3)
EPIs delivered annually, No.	
<10	1612 (57.2)
10-29	598 (21.2)
30-50	211 (7.5)
≥50	396 (14.1)

Abbreviation: EPI, extremely preterm infant.

### Attitudes of the Obstetricians Toward Resuscitation of EPIs at Different Gestational Ages and Birth Weights and Factors Associated With Resuscitation Decision of EPIs

The proportion of the 2817 obstetricians who supported active resuscitation for EPIs was 3.6% (102 respondents) for gestational age less than 24 weeks, 19.1% (539 respondents) at 24 weeks, 24.1% (679 respondents) at 25 weeks, 54.8% (1543 respondents) at 26 weeks, and 100.0% (2817 respondents) at 27 weeks. Of them, 2138 obstetricians (75.9%) maintained that 26 weeks or later should be the gestational age threshold for active resuscitation of EPIs (Table 2); 754 obstetricians (26.8%) supported active resuscitation regardless of birth weight of EPIs, 1095 (38.9%) supported resuscitation of EPIs with a birth weight of 500 to 749 g, 1746 (62.0%) supported resuscitation of EPIs with a birth weight of 749 to 999 g, and 2817 (100.0%) supported resuscitation of EPIs with a birth weight of 1000 g or more (Table 2). Gestational age was considered the most important factor associated with the resuscitation decision of EPIs, followed by parents’ willingness, the vitality at birth, birth weight, and antenatal corticosteroids, whereas gender was considered the least important factor (eFigure in the Supplement).

**Table 2.** Obstetricians’ Attitudes Toward Resuscitation of EPIs at Different Gestational Ages and Birth Weights

Items	Respondents, No. (%)	Cumulative, No. (%)
Supporting active resuscitation of EPIs at different gestational age, wk		
<22	50 (1.8)	50 (1.8)
22	29 (1.0)	79 (2.8)
23	23 (0.8)	102 (3.6)
24	437 (15.5)	539 (19.1)
25	140 (5.0)	679 (24.1)
26	864 (30.7)	1543 (54.8)
27	1274 (45.2)	2817 (100.0)
Supporting active resuscitation of EPIs at different birth weights, g		
Any weight	754 (26.8)	754 (26.8)
500-749	341 (12.1)	1095 (38.9)
750-999	651 (23.1)	1746 (62.0)
≥1000	1071 (38.0)	2817 (100.0)

Abbreviation: EPI, extremely preterm infant.

### Withdrawing Resuscitation

A total of 1326 obstetricians (47.1%) reported that it was very common (312 respondents [11.1%]) or common (1014 respondents [36.0%]) for the parents or families to withdraw their EPIs from resuscitation and to refuse to admit their EPIs to the NICU for treatment; 1945 obstetricians (69.0%) considered that the main reason for resuscitation withdrawal was the concern or apprehension about the poor prognosis, and only 624 (22.2%) thought that the main reason was associated with the family’s financial condition. A total of 1532 obstetricians (54.4%) felt uncertain about whether resuscitation of the EPIs should be implemented or continued, and 1772 (62.9%) felt hesitant because they had no confidence in the prognosis or worried about medical disputes with the parents due to poor prognosis of EPIs. If resuscitation of EPIs was unsuccessful, or prognosis of EPIs was poor, or EPIs with good birth conditions did not receive active resuscitation for some reasons, obstetricians would be involved in medical disputes with EPIs’ parents or families. The survey revealed that 989 obstetricians (35.1%) or their colleagues had been involved in litigation of medical disputes related to the resuscitation of EPIs.

Regarding the treatment of EPIs with good birth conditions and higher possibility of survival, 1621 obstetricians (57.5%) adopted an attitude of factual communication (ie, objective analysis of the situation, without obvious tendencies) but respecting parents’ opinions when their families requested nonresuscitation, 1121 (39.8%) recommended sending EPIs to the NICU for treatment, and only 75 (2.7%) followed the parents’ wishes when they requested nonresuscitation. As for the final decision on whether to treat EPIs, 1988 obstetricians (70.6%) maintained that the decision should be made by the parents, 646 (22.9%) held that it should be made by the neonatologists, and only 38 (1.3%) suggested that it should be made by the obstetricians. The results are shown in Table 3.

**Table 3.** Withdrawing Resuscitation and Care of EPIs

Items	Respondents, No. (%)
EPIs to be withdrawn from resuscitation by their parents	
Very common	312 (11.1)
Common	1014 (36.0)
Rare	1153 (40.9)
None	338 (12.0)
The main reason for withdrawing resuscitation	
Parents worry about prognosis	1945 (69.0)
High costs of treatment	624 (22.2)
Others	248 (8.8)
Obstetricians’ attitude when EPIs’ families request nonresuscitation	
Do as the parents wish	75 (2.7)
Actively persuade the parents to treat EPIs	1121 (39.8)
Factual communication, depends on parents	1621 (57.5)
Final decision maker of treatment for EPIs	
Parents	1988 (70.6)
Neonatologists	646 (22.9)
Obstetricians	38 (1.3)
Ethics committee	69 (2.4)
Others	76 (2.7)

Abbreviation: EPI, extremely preterm infant.

### Obstetricians’ Opinions About the Cutoff for Providing Full Care to Preterm Infants and Factors Associated With Their Opinions

Most obstetricians (2051 respondents [72.8%]) thought that the current gestational age (≥28 weeks) for providing full care to preterm infants was appropriate, and only 766 (27.2%) considered it inappropriate. Among the obstetricians who thought of it as inappropriate, 275 (35.9%) suggested that the lowest gestational age for providing full care to preterm infants should be 24 weeks or less or that no lowest gestational age should be set, and 390 (50.9%) suggested that lowest gestational age for providing full care to preterm infants should be 26 weeks or later (Figure).

**Figure.**
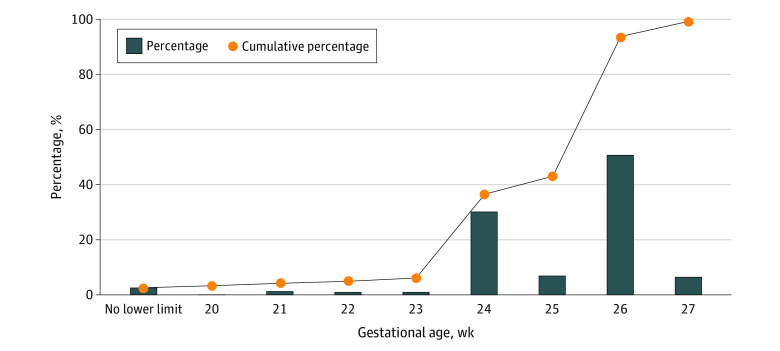
Obstetricians’ Opinions About the Appropriate Lowest Gestational Age for Providing Full Care to Preterm Infants

A multivariable logistic regression model was used to estimate factors associated with obstetricians’ opinions about the current cutoff for providing full care to preterm infants, and the results are shown in Table 4. The higher the professional title of the obstetricians, the longer the working years, the higher the hospital level, the more annual delivery of EPIs in their departments, the higher the proportion of the obstetricians who thought of the current gestational age for providing full care as inappropriate. In addition, there were significant regional differences in the attitude toward the treatment of EPIs, and the proportion of the obstetricians who thought of the current gestational age for providing full care to premature infants as inappropriate decreased in sequence in the eastern (reference), central (odds ratio [OR], 0.61; 95% CI, 0.48-0.78; *P* < .001), and western (OR, 0.44; 95% CI, 0.36-0.55; *P* < .001) regions. A total of 656 obstetricians (32.0%) with experience in delivering EPIs thought of the current gestational age for providing full care to preterm infants as inappropriate vs 110 obstetricians (14.3%) without experience (OR, 0.47; 95% CI, 0.36-0.60; *P* < .001). The proportion of obstetricians who considered the current cutoff for providing full care to premature infants inappropriate was higher in maternal and child health hospitals (OR, 1.42; 95% CI, 1.16-1.73; *P* = .001) than that in general hospitals.

**Table 4.** Factors Associated With Obstetricians’ Opinions About the Current GA for Providing Full Care to PIs

Variable	Respondents, No. (%)		OR (95% CI)	*P* value
	Current GA for providing full care to PIs is appropriate (n = 2051)	Current GA for providing full care to PIs is inappropriate (n = 766)		
Gender				
Male	94 (4.6)	62 (8.1)	1 [Reference]	.11
Female	1957 (95.4)	704 (91.9)	0.75 (0.52-1.07)	
Marital status				
Married	1848 (90.1)	709 (92.6)	1 [Reference]	NA
Unmarried	169 (8.2)	46 (6.0)	0.89 (0.51-1.55)	.67
Others	34 (1.7)	11 (1.4)	0.80 (0.39-1.65)	.55
Have children				
Yes	1798 (87.7)	690 (90.1)	1 [Reference]	.81
No	253 (12.3)	76 (9.9)	1.06 (0.66-1.72)	
Region				
Eastern	515 (25.1)	355 (46.3)	1 [Reference]	NA
Central	514 (25.1)	165 (21.5)	0.61 (0.48-0.78)	<.001
Western	1022 (49.8)	246 (32.1)	0.44 (0.36-0.55)	<.001
Professional title				
Resident physician	353 (17.2)	82 (10.7)	1 [Reference]	NA
Attending physician	687 (33.5)	217 (28.3)	1.18 (0.80-1.74)	.40
Deputy chief	681 (33.2)	227 (31.3)	1.56 (1.00-2.43)	.05
Chief	330 (16.1)	227 (29.6)	2.49 (1.52-4.08)	<.001
Working history, y				
<5	197 (9.6)	39 (5.1)	1 [Reference]	NA
5-9	285 (13.9)	103 (13.4)	1.41 (0.86-2.30)	.17
10-19	648 (31.6)	256 (33.4)	1.41 (0.81-2.46)	.22
20-29	646 (31.5)	248 (32.4)	1.04 (0.57-1.90)	.89
>30	275 (13.4)	120 (15.7)	0.89 (0.47-1.69)	.72
Hospital level				
Tertiary	1087 (53.0)	575 (75.1)	1 [Reference]	NA
Secondary	859 (41.9)	176 (23.0)	0.56 (0.45-0.70)	<.001
Primary	105 (5.1)	15 (2.0)	0.48 (0.27-0.87)	.02
Hospital category				
General	1513 (73.8)	520 (67.9)	1 [Reference]	.001
Maternal and child health care hospital	538 (26.2)	246 (32.1)	1.42 (1.16-1.73)	
Experience of delivering EPIs				
Yes	1392 (67.9)	656 (85.6)	1 [Reference]	<.001
No	659 (32.1)	110 (14.4)	0.47 (0.36-0.60)	
EPIs delivered annually, No.				
<10	1309 (63.8)	303 (39.6)	1 [Reference]	NA
10-29	394 (19.2)	204 (26.6)	1.56 (1.24-1.96)	<.001
30-50	125 (6.1)	86 (11.2)	2.13 (1.54-2.95)	<.001
>50	223 (10.9)	173 (22.6)	2.04 (1.57-2.65)	<.001

Abbreviations: EPI, extremely preterm infant; GA, gestational age; NA, not applicable; PI, preterm infant; OR, odds ratio.

## Discussion

Whether obstetricians are active in treating EPIs is directly associated with whether EPIs can get an opportunity to enter the NICU for treatment. To our knowledge, this is the first nationwide, cross-sectional, online, questionnaire-based survey on obstetricians’ attitudes toward the treatment of EPIs, covering 30 provinces and cities in mainland China. We found that most obstetricians in China had a conservative attitude toward the treatment of EPIs, which was mainly associated with the concerns about their poor prognosis.

Because of the difficulty of treatment and the high incidence of complications after survival, it has always been a clinical dilemma whether to actively resuscitate EPIs, especially those in the gray area below 25 weeks.^14,15^ At present, with the advancement of treatment technology, developed countries have a more positive attitude toward the treatment of EPIs, especially for EPIs with a gestational age of 24 weeks or more. A survey conducted in the UK, Sweden, and the Netherlands showed that, for an EPI who was born in a good condition, regarding the lowest gestational age at which resuscitation would be provided, 60% of UK respondents advocated resuscitation beyond 23 weeks’ gestational age, 56% of Swedish respondents advocated resuscitation beyond 22 weeks’ gestational age, and 58% of Dutch respondents advocated resuscitation beyond 24 weeks’ gestational age.^16^ A Dutch study^17^ reported that 54% and 64% respondents advocated implementation of intensive care for infants at 24 and 25 weeks’ gestation, respectively, and 89% and 96% of respondents advocated implementation of resuscitation for infants at 26 and 27 weeks’ gestation, respectively. A Japanese study^18^ suggested that the limit of viability of EPIs should be 22 weeks’ gestational age, and approximately 50% of EPIs were estimated to receive active resuscitation. At present, the enthusiasm for treating EPIs in China is obviously lower than that in developed countries. A study in Shandong Province of China^13^ reported that 73% of the 1163 EPIs with a gestational age of 24 to 27 weeks were withdrawn from resuscitation in the delivery room, and the overall survival rate was 20.7%, indicating that most EPIs did not receive aggressive resuscitation and NICU treatment. The data of our study showed that few obstetricians investigated supported resuscitation for EPIs with a gestational age of less than 26 weeks. Compared with developed countries, the enthusiasm for treating EPIs in China is by far lower.

In China, the cutoff for providing full care to premature infants and the starting point of the perinatal period both have long been specified as 28 weeks, and withdrawal of care is common for EPIs. However, little attention has been paid to whether this cutoff for providing full care is rational and appropriate in the context of remarkable advancements in the fields of gynecology and obstetrics in recent decades, knowing that most developed countries have renewed the minimum gestational age for perinatal death registration to about 20 weeks.^19^ The cutoff for providing full care to premature infants also directly affects the statistics of neonatal mortality in China. In recent years, the treatment capacity of EPIs in China has rapidly improved, and the survival rate has increased from 45.1% in 2008 to 2012^20^ to 68.0% in 2010 to 2019^11^ for infants born at 24 to 27 weeks. In addition, with China’s economy developing, social and economic pressures on families are increasing, and young people choose not to have children or only 1 child. Although it is advocated for a couple to have 2 children, the status quo has not improved very much. At present, the birth rate in China has sharply declined, and China is gradually entering an aging society. It is very important to actively treat every born child, and it is imperative to pay attention to the treatment of EPIs. However, according to the data of the present survey, only a minority (27.2%) of the obstetricians investigated thought that the currently existing cutoff for providing full care to premature infants was inappropriate and should be brought down. Among them, 50.9% still suggested that the lowest gestational age for providing full care to premature infants should be 26 weeks or later, and a tiny minority suggested that the cutoff should be 24 weeks or less, or that no lowest limit should be set. These different opinions on the whole reflect that obstetricians in China still hold a conservative attitude toward the treatment of EPIs.

It was also found in the present study that the obstetricians’ attitudes toward treatment of EPIs were associated with multiple factors. Obstetricians with more experience in delivering EPIs, higher professional title, longer working years, and those working in higher level hospitals and departments with more EPIs delivered were more inclined to think that the current gestational age for providing full care to premature infants was inappropriate. This suggests that the more contacts of the obstetricians with EPIs, the more positive attitude they would have toward treatment. However, another study^21^ reported that physicians’ years of experience did not have a significant impact on their attitude toward EPI resuscitation. A survey in a developing country^22^ found that physicians aged 46 to 55 years and those older than 55 years were more likely to choose a higher birth weight as the limit of viability, and similar results were observed among physicians with 10 to 20 years of practice and those with more than 20 years of practice. The possible reason for obstetricians’ positive attitude toward the treatment of EPIs in our study is that they come from large-scale tertiary hospitals, where the survival rate of EPIs is high and the incidence of sequelae is low, so they are more confident in the treatment of EPIs.

In China, economic development is not regionally balanced: the eastern region is the most economically developed, followed by the central region, and the western region is the least economically developed. The same is true of medical development. Regional distribution is also a factor affecting the attitude toward treatment of EPIs, so the enthusiasm of obstetricians in treating EPIs decreased successively in the eastern, central, and western regions. This is completely consistent with the regional difference in distribution of the neonatal treatment resources reported in our previous investigation.^23^ This also indicates that the ability and level of treating EPIs directly affect the treatment attitude toward EPIs. This is consistent with the results of a Lebanese study,^22^ which found that health care resource allocation was ranked second as an influencing factor of treatment of EPIs, and physicians practicing in higher level NICUs (levels ≥3) had a significant trend of choosing lower birthweight limit of viability compared with those practicing at a facility with no NICU.

Although most obstetricians investigated in China prescribed antenatal corticosteroids to EPIs and worked together with the pediatricians in birth attendance of EPIs, they on the whole held a negative attitude toward the treatment of EPIs. It was found in our study that more than half of the obstetricians felt uncertain whether EPIs should be sent to the NICU for treatment, mainly because they were not sure about the prognosis of EPIs or worried about the emergence of disputes in case a poor prognosis should occur. Because of the lack of relevant laws in this field, medical and ethical disputes arising from EPIs will surely increase in future and therefore relevant legal and ethical systems need to be augmented urgently.

This study found that the obstetricians’ attitude toward the treatment of EPIs remains dilemmatic in China at present. In addition, the attitudes toward the treatment of EPIs are closely associated with the experience of the obstetricians concerned and their ability to deal with EPIs. With the further decline of the birth rate and the trend of having fewer children in the family, the treatment of EPIs is bound to arouse more attention. In our opinion, strengthening the collaboration between obstetrics and pediatrics, improving the treatment level of EPIs and the prognosis, and establishing sound legal and ethical systems should be given top priority in the future work in this field in China.

### Limitations

This study has some limitations. First, it is a cross-sectional study and the participants were not selected randomly, which may limit generalizability. Second, the number of participants varies among provinces, which may result in a potential sampling bias.

## Conclusions

The results of this nationwide, cross-sectional, online, questionnaire-based survey provide evidence that most Chinese obstetricians maintain a conservative attitude toward the treatment of EPIs. It is very common for EPIs to be withdrawn from treatment without entering the NICU directly after birth. Most obstetricians think that the lowest gestational age for providing full care to premature infants should be 28 weeks.

## Notes

Supplement. **eFigure.** Factors Influencing Resuscitation Decision of EPIs

## References

[1] Shukla A, Beshers C, Worley S, Chowdhary V, Collin M. In the grey zone: survival and morbidities of periviable births. J Perinatol. 2022;42(8):1001-1007. doi:10.1038/s41372-022-01355-z 35273353

[2] Blencowe H, Cousens S, Chou D, . Born too soon: the global epidemiology of 15 million preterm births. Reprod Health. 2013;10(suppl 1):S2. doi:10.1186/1742-4755-10-S1-S2 24625129 PMC3828585

[3] Brunkhorst J, Weiner J, Lantos J. Infants of borderline viability: the ethics of delivery room care. Semin Fetal Neonatal Med. 2014;19(5):290-295. doi:10.1016/j.siny.2014.08.001 25153263

[4] Salama H, Al Rifai H, Mahmoud N, . Selection criteria for resuscitation and survivability rates for neonates at the limit of viability. J Neonatal Perinatal Med. 2020;13(2):153-158. doi:10.3233/NPM-190249 31744024

[5] Stoll BJ, Hansen NI, Bell EF, ; Eunice Kennedy Shriver National Institute of Child Health and Human Development Neonatal Research Network. Trends in care practices, morbidity, and mortality of extremely preterm neonates, 1993-2012. JAMA. 2015;314(10):1039-1051. doi:10.1001/jama.2015.10244 26348753 PMC4787615

[6] Kaempf J, Morris M, Steffen E, Wang L, Dunn M. Continued improvement in morbidity reduction in extremely premature infants. Arch Dis Child Fetal Neonatal Ed. 2021;106(3):265-270. doi:10.1136/archdischild-2020-319961 33109606

[7] Cavolo A, Dierckx de Casterlé B, Naulaers G, Gastmans C. Physicians’ attitudes on resuscitation of extremely premature infants: a systematic review. Pediatrics. 2019;143(6):e20183972. doi:10.1542/peds.2018-3972 31076541

[8] Bell EF, Hintz SR, Hansen NI, ; Eunice Kennedy Shriver National Institute of Child Health and Human Development Neonatal Research Network. Mortality, in-hospital morbidity, care practices, and 2-year outcomes for extremely preterm infants in the US, 2013-2018. JAMA. 2022;327(3):248-263. doi:10.1001/jama.2021.23580 35040888 PMC8767441

[9] Ma L, Liu C, Cheah I, . Cost is an important factor influencing active management of extremely preterm infants. Acta Paediatr. 2019;108(1):70-75. doi:10.1111/apa.14533 30080290

[10] Collaborative Study Group for Extremely Preterm and Extremely Low Birth Weight Infants. Short-term outcomes and their related risk factors of extremely preterm and extremely low birth weight infants in Guangdong province [in Chinese]. Zhonghua Er Ke Za Zhi. 2019;57(12):934-942. doi:10.3760/cma.j.issn.0578-1310.2019.12.008 31795560

[11] Zhu Z, Yuan L, Wang J, . Mortality and morbidity of infants born extremely preterm at tertiary medical centers in China from 2010 to 2019. JAMA Netw Open. 2021;4(5):e219382. doi:10.1001/jamanetworkopen.2021.9382 33974055 PMC8114138

[12] Obstetrics Group, Obstetrics and Gynecology Branch of Chinese Medical Association. Guidelines for clinical diagnosis and treatment of premature infants [in Chinese]. Chinese J Perinat Med. 2015;18(4):241-245. doi:10.3760/cma.j.issn.0529-567x.2015.04.001.001

[13] Zhang WW, Yu YH, Dong XY, Reddy S. Treatment status of extremely premature infants with gestational age < 28 weeks in a Chinese perinatal center from 2010 to 2019. World J Pediatr. 2022;18(1):67-74. doi:10.1007/s12519-021-00481-6 34767193 PMC8761149

[14] Cavolo A, Dierckx de Casterlé B, Naulaers G, Gastmans C. Ethics of resuscitation for extremely premature infants: a systematic review of argument-based literature. J Med Ethics. 2020;47:e4. doi:10.1136/medethics-2020-106102 32341186

[15] Weir M, Evans M, Coughlin K. Ethical decision making in the resuscitation of extremely premature infants: the health care professional’s perspective. J Obstet Gynaecol Can. 2011;33(1):49-56. doi:10.1016/S1701-2163(16)34773-9 21272437

[16] Wilkinson D, Verhagen E, Johansson S. Thresholds for resuscitation of extremely preterm infants in the UK, Sweden, and Netherlands. Pediatrics. 2018;142(suppl 1):S574-S584. doi:10.1542/peds.2018-0478I 30171144 PMC6379058

[17] Geurtzen R, Draaisma J, Hermens R, . Perinatal practice in extreme premature delivery: variation in Dutch physicians’ preferences despite guideline. Eur J Pediatr. 2016;175(8):1039-1046. doi:10.1007/s00431-016-2741-7 27251669 PMC4930484

[18] Isayama T. The clinical management and outcomes of extremely preterm infants in Japan: past, present, and future. Transl Pediatr. 2019;8(3):199-211. doi:10.21037/tp.2019.07.10 31413954 PMC6675688

[19] Barfield WD, Watterberg K, Benitz W, ; Committee on Fetus and Newborn. Standard terminology for fetal, infant, and perinatal deaths. Pediatrics. 2016;137(5):e20160551. doi:10.1542/peds.2016-0551 27244834

[20] Collaborative Study Group for Extremely Preterm & Extremely Low Birth Weight Infants. Survival and mortality rate of extremely preterm and extremely low birth weight infants admitted to neonatology departments [in Chinese]. Zhonghua Er Ke Za Zhi. 2014;52(10):729-735. 25537536

[21] Kunkel MD, Downs SM, Tucker Edmonds B. Influence of maternal factors in neonatologists’ counseling for periviable pregnancies. Am J Perinatol. 2017;34(8):787-794. doi:10.1055/s-0037-1598247 28192814

[22] Charafeddine L, Ammous F, Kayle M, Arawi T. Survival at the threshold of viability: a nationwide survey of the opinions and attitudes of physicians in a developing country. Paediatr Perinat Epidemiol. 2014;28(3):227-234. doi:10.1111/ppe.12118 24654779

[23] Li Q, Li X, Zhang Q, . A cross-sectional nationwide study on accessibility and availability of neonatal care resources in hospitals of China: current situation, mortality and regional differences—neonatal care resources and newborn mortality in China. Lancet Reg Health West Pac. 2021;14:100212. doi:10.1016/j.lanwpc.2021.100212 34528000 PMC8358159

