# Supplementary material for: Obstetricians’ Attitudes Toward the Treatment of Extremely Preterm Infants in China
Source: JAMA Netw Open. 2022 Sep 27;5(9):e2233511. doi: 10.1001/jamanetworkopen.2022.33511 (PMC9516285; doi:10.1001/jamanetworkopen.2022.33511)
Supplement: Supplement. — eFigure. Factors Influencing Resuscitation Decision of EPIs [file jamanetwopen-e2233511-s001.pdf]

Supplemental Online Content

Han T, Wang D, Xie W, et al. Obstetricians' attitudes toward the treatment of extremely preterm infants in China. *JAMA Netw Open*. 2022;5(9):e2233511. doi:10.1001/jamanetworkopen.2022.33511

eFigure. Factors Influencing Resuscitation Decision of EPIs

This supplemental material has been provided by the authors to give readers additional information about their work.

© 2022 Han T et al. *JAMA Network Open.*

eFigure. Factors Influencing Resuscitation Decision of EPIs

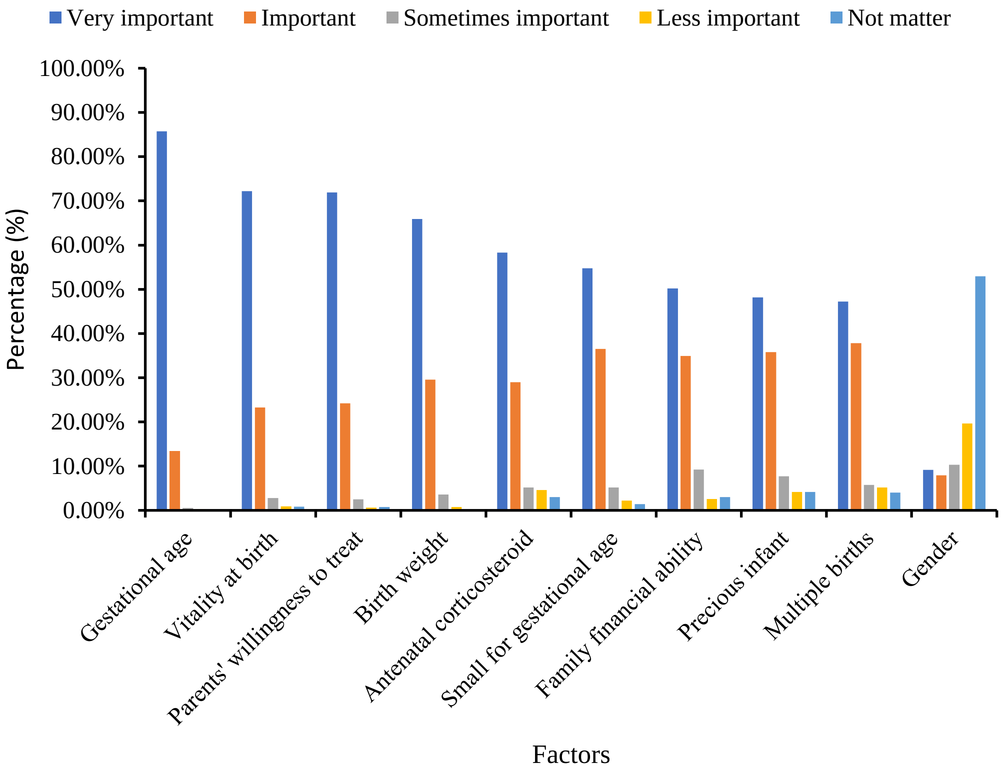

© 2022 Han T et al. *JAMA Network Open.*
